# Performance Deficits in a Voluntary Saccade Task in Chinese “Express Saccade Makers”

**DOI:** 10.1371/journal.pone.0047688

**Published:** 2012-10-16

**Authors:** Paul C. Knox, Nabin Amatya, Xiaoyu Jiang, Qyong Gong

**Affiliations:** 1 Eye and Vision Science, Institute of Ageing and Chronic Disease, University of Liverpool, Liverpool, United Kingdom; 2 Department of Radiology, Centre for Medical Imaging, Huaxi MR Research Centre, West China Hospital, Sichuan University, Chengdu, China; University of Muenster, Germany

## Abstract

Differences in behaviour and cognition have been observed in different human populations. It has been reported that in various types of complex visual task, eye movement patterns differ systematically between Chinese and non-Chinese participants, an observation that has been related to differences in culture between groups. However, we confirm here that, in healthy, naïve adult Chinese participants, a far higher proportion (22%) than expected (1–5%) exhibit a pattern of reflexive eye movement behaviour (high numbers of low latency express saccades) in circumstances designed to inhibit such responses (prosaccade overlap tasks). These participants are defined as “express saccade makers” (ESMs). We then show using the antisaccade paradigm, which requires the inhibition of reflexive responses and the programming and execution of voluntary saccades, that the performance of ESMs is compromised; they have higher antisaccade directional error rates, and the latency distributions of their error saccades again exhibit a higher proportion of low latency express saccade errors consistent with a reduced ability to inhibit reflexive responses. These results are difficult to reconcile with a cultural explanation as they relate to important and specific performance differences within a particular population. They suggest a potential unexpected confound relevant to those studies of Chinese versus other groups which have investigated group differences using oculomotor measures, and explained them in terms of culture. The confirmation of higher numbers of ESMs among Chinese participants provides new opportunities for examining oculomotor control.

## Introduction

Over the last decade, cognitive and behavioural differences between human populations have been reported in a wide range of studies [1], [2], [3], [4], [5], [6]. It has recently also been argued that most of what is currently known about human cognition is based on data from a strikingly unrepresentative sample of the global human population [7]. So on the one hand the literature is dominated by data generated from this narrow participant base, while on the other when different populations are examined it appears that brain and behaviour vary systematically for reasons that remain a matter of debate [8], [9].

A useful contrast might be drawn between differences in cognition (eg in processes such as memory and attention) and differences in reflexive behaviours. We recently demonstrated a difference between groups of Chinese and (white) UK participants in a reflexive saccade task [10]. Express saccades (ES) are low latency visually-guided saccades that have a distinct neurophysiological origin [11], [12], [13]. Although saccade latency is modified by many factors, and is dependent on task design, saccades with latency in the range of 80 ms to 130 ms can reasonably be considered to be ES [10], [14]. In circumstances which greatly decrease the occurrence of express saccades – prosaccade overlap tasks in which a central fixation target remains present when the saccade target appears - we found that 29% of Chinese participants persisted in producing high numbers of ES compared to only 3% of the UK group. This phenomenon has been reported previously [15], [16], giving rise to the concept of the “express saccade maker” (ESM), a naïve participant who in the absence of any pathology exhibits a high proportion (>30%) of ES in overlap conditions. A previous informal estimate suggested that ESMs are encountered rarely (comprising 1% to 5% of the adult population [15]); this is consistent with a number of relatively large saccade studies that did not observe any ESMs [17], [18].

Confirmation of high numbers of ESMs among Chinese participants would provide a new avenue for the investigation of population differences as well as a means of investigating ESMs in greater numbers than previously possible. It would also imply a possible confound in experiments which have found differences in eye movement patterns between Chinese and non-Chinese groups and attributed them to culture [19], [20]. Where dependant variables such as fixation time or saccade number have been the focus of analysis, altered low-level oculomotor performance in the Chinese group might explain part or all of the group differences in performance. What we do not yet know is whether the saccade performance differences in Chinese participants generalise to tasks beyond the simple reflexive tasks that we used previously.

The links between saccade behavioural measurements and their underlying neurophysiology are well understood. Therefore, saccades provide a behavioural means of investigating specific neural circuits [21]. If in ESMs there is an important general alteration in oculomotor processing, then their performance should be distinguishable from non-ESM participants across testing paradigms. And their performance in other task types should provide important additional information allowing stronger inferences to be made about which specific aspects of the saccade system (in both functional and anatomical terms) are altered in ESMs. Finally, if ESMs, defined on the basis of their performance on reflexive saccade tasks, do perform differently from non-ESM participants in other task types, this would add validity to their classification as a distinct group.

We therefore tested a large group of naïve Chinese participants in order to identify ESMs, and to confirm whether ESMs occurred in larger numbers than expected. We then used the antisaccade task [22], [23], which requires the participant to inhibit a reflexive saccade towards a target, and compute and execute a voluntary saccade to the mirror image of the target position, to explore their oculomotor control further. Directional errors, composed of error prosaccades (saccades towards the target), reflect problems inhibiting reflexive responses. Correct antisaccade latency is considerably greater than the latency of error prosaccades, because of both the processing required to inhibit the reflexive response and to compute the appropriate voluntary saccade. Antisaccade task performance is critically dependent on a number of cortical areas, particularly the frontal eye fields (FEF) and dorsolateral prefrontal cortex (dlPFC) [23].

## Materials and Methods

### Ethics Statement

Experiments were specifically approved by the West China Hospital of the University of Sichuan Ethics Committee. All participants provided their written informed consent and experiments were performed in accordance with the ethical standards laid down in the Declaration of Helsinki (as modified 2004).

### Participants

A total of 77 healthy, naïve, adult participants with normal or corrected to normal visual acuity were recruited from staff and students of, and tested in, the West China Hospital, Chengdu, China. The median age of the group was 24 y (range 19 y–45 y), and 39 (50%) were male. All were Han Chinese.

### Apparatus and Stimuli

Horizontal eye movements were recorded binocularly with the same miniaturized head-mounted infrared saccadometer (Advanced Clinical Instrumentation, Cambridge, UK) used in our previous experiment [10]. This samples infrared reflectance signals at 1 KHz, and low-pass filters them at 250 Hz with 12-bit resolution. The device incorporates three low-power red lasers projecting 13 cd/m^2^ target spots subtending approximately 0.1°, in a horizontal line, centrally and at 10° to left and right of centre. As the stimuli move with the head, participants were not head-fixed; they sat in a comfortable position approximately 1.5 m in front of a near-white surface.

We exposed participants to two blocks of trials of two types: prosaccade overlap and synchronous antisaccade trials. Each block consisted of two runs of 200 trials (thus we had a potential maximum of 400 trials of each type). In prosaccade trials after a randomised fixation time of 1 s–2 s, a saccade target appeared randomly 10° to the right or left of the central fixation target while the fixation target remained illuminated. Participants were instructed to saccade to the eccentric target as soon as they detected it. These tasks were identical to the prosaccade overlap tasks that we used previously [10]. In antisaccade trials a synchronous task was used in which after the same variable fixation time the fixation target was extinguished and a saccade target appeared randomly 10° to the right or left. Participants were instructed not to look at the target, but to saccade to its mirror image position ie 10° from the central fixation target, in the opposite direction to the target. At the beginning of each run we stepped through the task while giving verbal instructions. We asked participants to respond as quickly and accurately as they were able, and during the antisaccade runs provided verbal reminders to “look in the opposite direction from the target, but the same distance from fixation”. The order of the two prosaccade and antisaccade blocks was counterbalanced across participants.

### Analysis

Data were stored on the Saccadometer handset, and downloaded for offline analysis using the supplied software (Latency Meter 4.0). The latency and amplitude of each prosaccade from the overlap blocks were collated and saccade latency distributions calculated for each individual participant. We excluded from the analysis saccades with a latency of less than 50 ms or more than 500 ms. Median latency and mean saccade amplitude were calculated for each participant. We also calculated the percentage of express saccades, that is those saccades with latency in the range 80 ms to 130 ms [14], [24]. We defined participants who had greater than 30% of their saccades in this range as “express saccade makers” (ESMs [10], [15]). For each participant we calculated antisaccade directional error rate (ie the number of saccades towards the target in antisaccade tasks, as a percentage of the total), median prosaccade error latency, and median antisaccade latency (for all saccades with latency between 50 ms and 700 ms). For each participant the distribution of these latencies was also calculated. We also calculated mean saccade amplitude for prosaccade errors and correct antisaccades.

## Results

### Prosaccade Tasks

**Figure 1:**
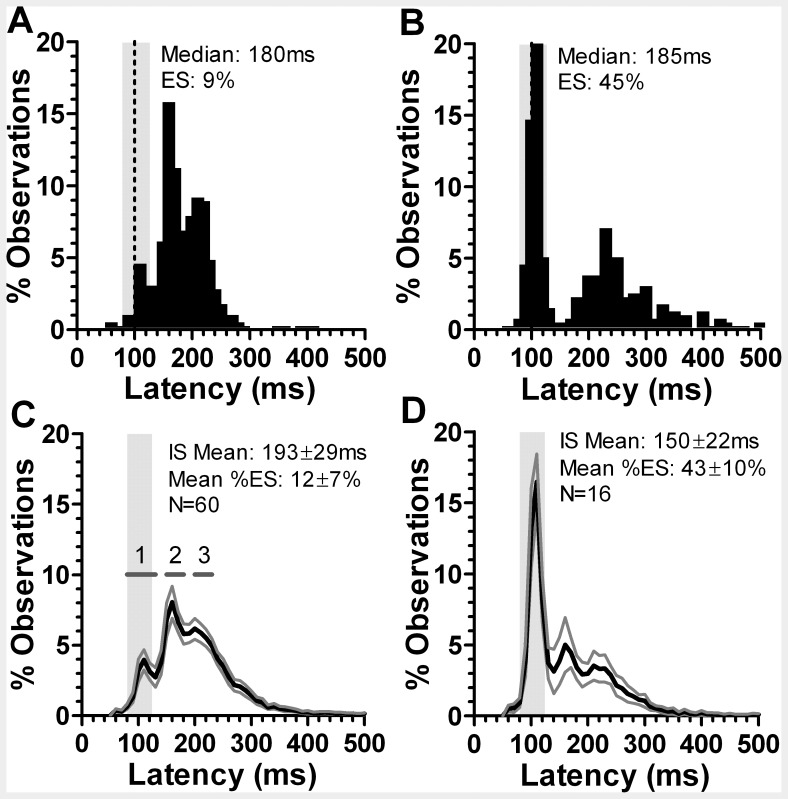
Percentage frequency distribution histograms of saccade latency in the prosaccade overlap task. A. Example from an individual “normal” subject; B. individual ESM. In A. and B. the median saccade latency, and the percentage of express saccades is shown. C. Mean±95%CI distribution for 60 “normal” subjects. D. Mean±95%CI distribution for 16 ESMs. In C. and D. the intersubject mean (±SD) of the individual median latencies and the intersubject mean (±SD) percentage of express saccades is shown. The vertical grey region shows the range of express saccade latency (80 ms to 130 ms) and the dotted vertical line is at 100 ms. The three horizontal grey bars in C. show the three latency ranges (1 to 3) over which latency was compared between ESMs and normal subjects.

**Figure 2:**
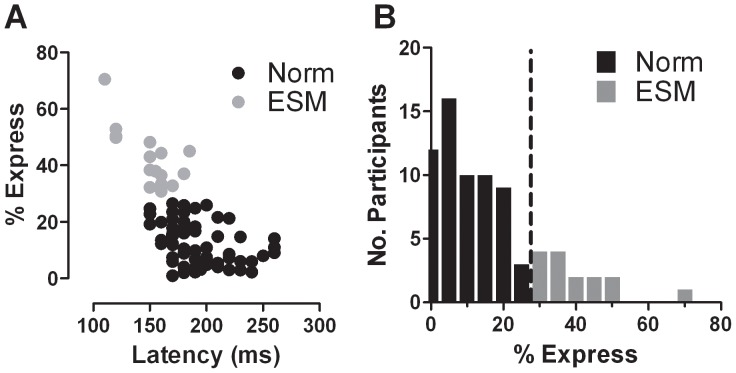
Data from prosaccade task. A. Plot of the percentage of express saccades against median prosaccade latency. B. Distribution of percentage of express saccades in the prosaccade task. Vertical dashed line shows the criterion used to define an ESM (30% ES in the prosaccade task). Columns to the right of this line show counts of ESMs.

Prosaccade overlap data were obtained for all 77 participants. The intersubject mean median saccade latency across all 77 was 184±32 ms. However, we observed a high number of individual frequency distributions histograms (Figure 1A,B) in which there was a clear early latency peak, centred close to 100 ms (Figure 1B; all the individual frequency distributions histograms are shown in Supporting Information as Figures S1 and S2). In 17 of the 77 participants (22%), the proportion of saccades with latency in the range 80 ms to 130 ms (express saccades, ES) exceeded 30% (Figure 2). We defined these 17 participants as “express saccade makers” (ESMs) and the remaining participants as “normal”. Mean median latency and percentage of ES for the ESMs were 150±22 ms and 43±10% compared to 193±28 ms and 12±7% for the 60 normals respectively. Both latency (t = 5.67; p<0.0001) and percentage of express saccades (t = 13.37; p<0.0001) were statistically significantly different between these two groups.

For each group, the individual percentage distribution histograms for each participant were used to calculate the mean (±95% CI) percentage for each 10 ms histogram bin in order to construct a mean distribution histogram (Figure 1C,D). This analysis confirmed a group difference in latency distributions, with the most noticeable feature being the early peak in the ESM distribution centred at around 100 ms. However, this analysis also suggested that this early peak in the ESM distribution was complemented by later peaks at 160 ms and 210 ms where the percentages were greater in the normal compared to the ESM distribution. We compared the mean bin values across the ranges 80 ms to 130 ms (the express saccade range; Figure 1C, range 1), 150 ms to 180 ms (range 2), and 200 ms to 230 ms (range 3) between the two groups. For each range the data were analysed with a repeated measures ANOVA, treating “latency bin” as a within subjects factor and group (normal vs ESM) as a between subjects factor. For all three ranges the effect of group was statistically significant (F_1,74_ = 577, 153 and 233 respectively, all p<0.001).

### Antisaccade Tasks

**Figure 3:**
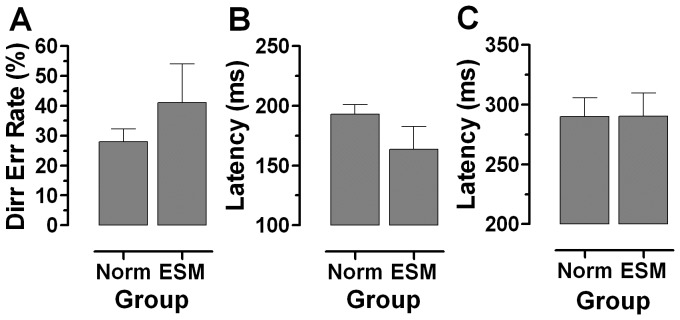
Data from antisaccade tasks. Comparison of mean±95%CI between 60 normal (non-ESM) subjects and 16 ESMs. A. Antisaccade directional error rate. B. Mean error pro-saccade latency. C. Mean correct antisaccade latency. Note different y-axis scales in B and C.

Of the 17 ESM participants, antisaccade data were obtained for 16. Subsequent analysis is based on this group of 16 ESMs (median age 24 y, 7 males) compared to a group of 60 normal participants (median age 23.5 y, 30 males). ESM antisaccade directional error rate (41±24%; Figure 3A) was statistically significantly higher (t = 2.5, p = 0.01) compared to the normal participants (28±16%). Error pro-saccade latency (ie saccades directed at the target in the antisaccade task, ErrPS) and correct antisaccade latency (CorAS) were analysed. For all bar one of the normal participants, and all of the ESMs, median ErrPS latency was less than that of CorAS latency. However, the intersubject mean difference between the medians (CorAS-ErrPS) was 97±54 ms and 127±45 ms for the normal and ESM groups respectively; this difference was statistically significant (t = 2.01, p<0.05). The reason for this greater difference in the EMSs was that while CorAS latency was identical between the groups (Figure 3C; Norm: 290±61 ms vs ESM:290±37 ms), the ErrPS latency was lower in the ESMs (Figure 3B; Norm:193±31 ms vs ESM: 164±35 ms). When investigated with a repeated measures ANOVA with saccade type (ErrPS vs CorAS) as a within subjects factor, and group (Norm vs ESM) as a between subjects factor, saccade type was significant (F_1,74_ = 233.9, p<0.001) while group was not (F_1,74_ = 1.9, p = 0.187). The interaction between type and group was statistically significant (F_1,74_ = 4.1, p = 0.045).

**Figure 4:**
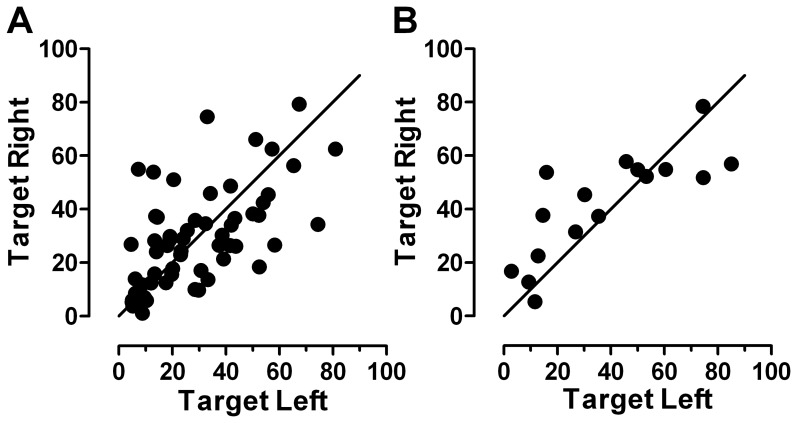
Influence of target direction (Left vs Right) on the antisaccade directional error rate (%) for A. Normal participants; B ESMs. Solid line is the line of equality (x = y).

We examined the influence of target direction on the generation of errors by calculating the directional error rate separately for each participant in each direction (Figure 4). Note that by target direction we are referring to the side on which the visual target was presented, not the required direction of a correct antisaccade. For normal participants there was very little difference in the rightward (29±19%) and leftward (20±20%) error rates, with 29/60 participants (48%) having a larger absolute error rate for rightward targets. For the ESMs there was more evidence of an asymmetry with the rightward mean error rate (42±20%) slightly higher than the leftward (38±26%); 11/16 (69%) exhibited higher error rates when targets were presented on the right.

**Figure 5:**
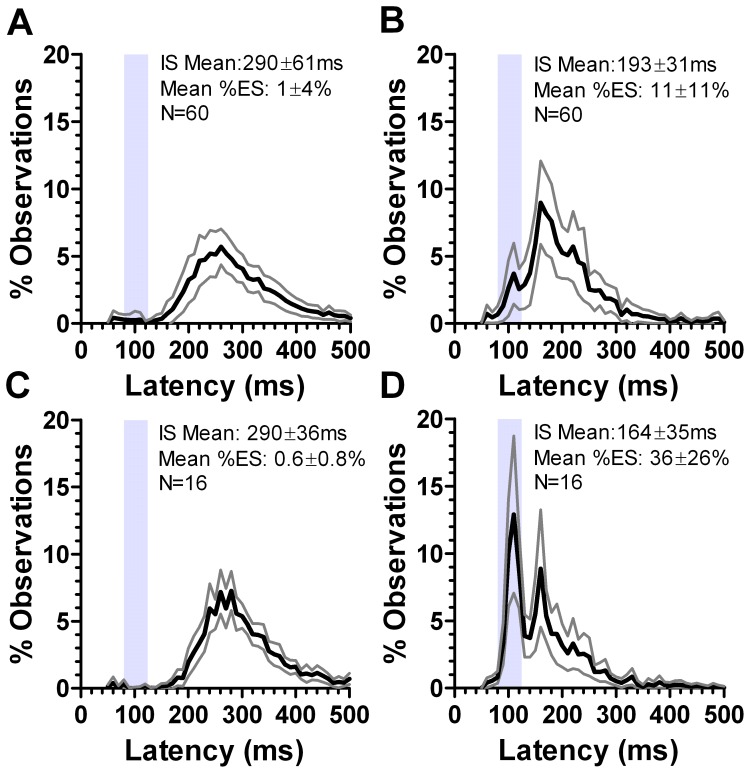
Mean±95%CI distributions for 60 normal subjects (A,B) and 16 ESMs (C,D) for correct antisaccades (A,C) and error prosaccades (B,D). The grey region shows the express saccade latency range (80 ms to 130 ms). The intersubject mean of the individual subject median latencies (±SD), and the intersubject percentage of express saccades is also shown.

We examined the distribution of CorAS and ErrPS latencies in the two groups by plotting mean (±95% CI) distribution histograms for each saccade type in each of the groups (Figure 5). While the distribution of CorrAS latency was identical, the lower mean latency for ESM ErrPS latency was explained by a prominent early peak in the distribution, which (as in overlap tasks) occurred in the express saccade range. The mean proportion of ErrPS with latencies in the express range was 36±26%. This contrasted with the normal group in whom there were fewer errors with latency in the express range (11±11%). The difference between these percentages was statistically significant (t = 5.69, p<0.0001).

### Relationship between Overlap and Antisaccade Performance

**Figure 6:**
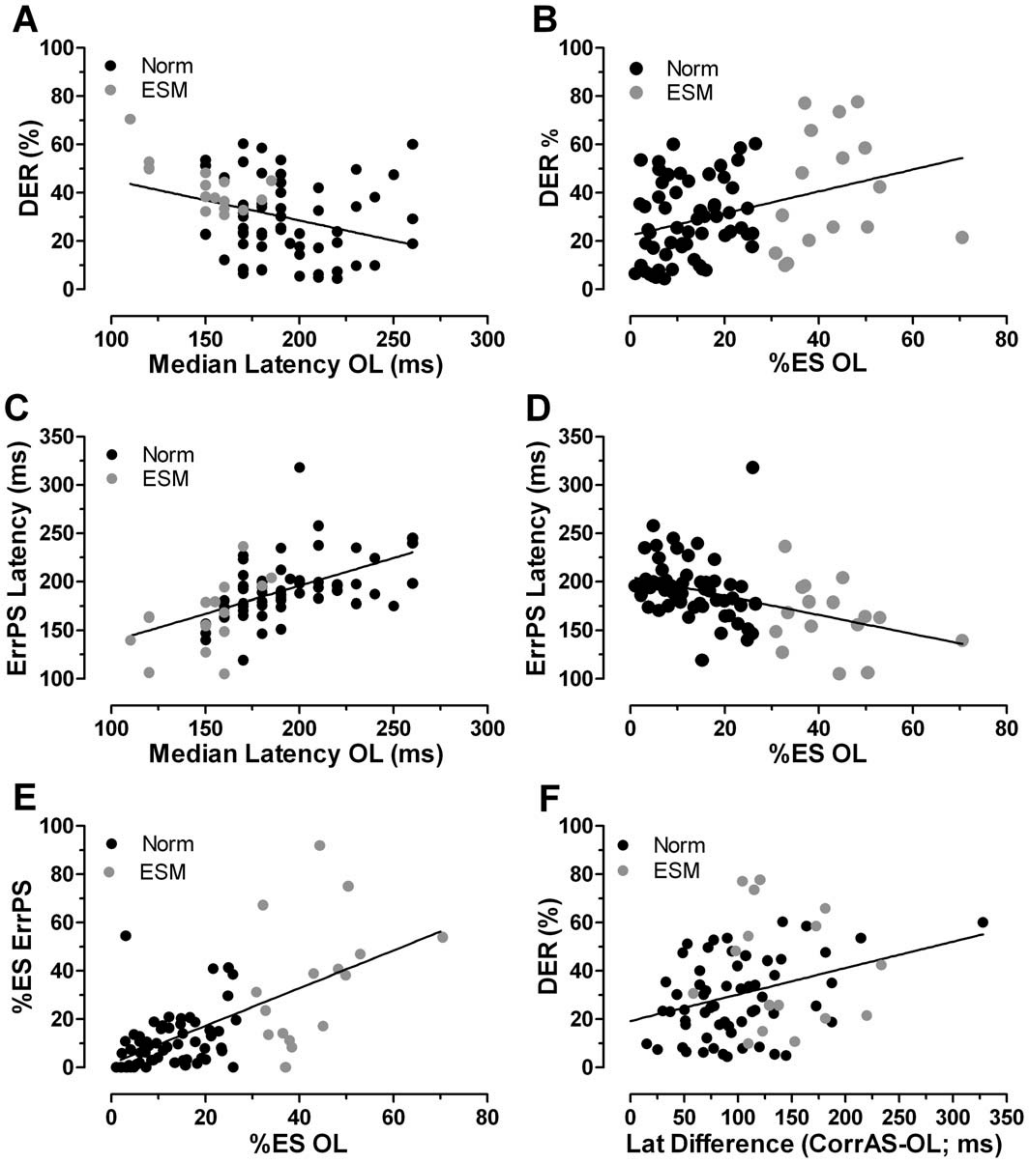
Relationship between prosaccade (PS) and antisaccade (AS) performance. A. Median prosaccade latency and AS directional error rate. B. Percentage of express saccades in the PS task and AS directional error rate. C. Median PS latency and median AS prosaccade error latency. D. Percentage of express saccades in the PS task and median AS error prosaccade latency (AS). E. Percentage of express saccades in the PS task and percentage of ES in AS prosaccade errors. F. The difference between correct AS latency and PS latency calculated for each subject, and AS directional error rate. On each plot the solid black line is the least-squares linear regression line calculated for the whole dataset. Data from ESMs: grey symbols; data from normal subjects: black symbols.

The two groups, defined on the basis of their performance on the prosaccade task, exhibited different patterns of performance in the antisaccade task. What then of the relationship between performance in the two different tasks? Performance in the prosaccade task was summarised using median saccade latency for each participant and the percentage of express saccades. We investigated the relationship between these parameters and antisaccade directional error rate (expressed as a percentage) and median ErrPS latency (Figure 6). Antisaccade directional error rate was correlated both with the median latency in the prosaccade task (Figure 6A; r = −0.33, p = 0.003) and with the percentage of express saccades (6B; r = 0.36, p = 0.002). Correlation coefficients were slightly higher between the median prosaccade latency and antisaccade error prosaccade latencies (6C; r = −0.55, p<0.0001) and percentage of express saccades in prosaccade tasks and antisaccade prosaccade error latency (6D; r = 0.43, p<0.0001). The highest correlation coefficient was observed for the relationship between the percentage of express saccades in prosaccade tasks, and the percentage of ErrPS that were express saccades (Figure 6E; r = 0.63, p<0.0001). We also examined whether a general difference in the speed of prosaccade and antisaccade systems might have a strong influence on directional error rate. For each participant we calculated the difference between the median correct antisaccade latency and the median prosaccade latency, and plotted this against the antisaccade directional error rate (Figure 6f). While there was a positive correlation, it was modest (r = 0.32, p = 0.004).

## Discussion

Our first objective was to establish the proportion of express saccade maker participants (ESMs) in a large group of healthy, adult, naïve Chinese participants using a prosaccade overlap task, identical to that used in our previous study [10]. In such tasks, the central fixation target remains illuminated when the eccentric saccade target appears. Compared to prosaccade gap tasks, the continued presence of the fixation target provides no visual warning signal of the imminent appearance of the saccade target caused by early fixation offset, and makes fixation disengagement more difficult. This usually results in an increase in saccade latency and a reduction in express saccades [24], [25].

Previous estimates [15] and studies in which relatively large numbers of participants had been tested [17], suggest that ESMs comprise no more than 5% of healthy adults. In our earlier experiment [10] 29% of a naïve Chinese group were ESMs compared to 3% in a UK group. In the current study 17/77 (22%) were ESMs. Thus ESMs were again encountered much more frequently than expected.

We have used the criterion employed previously [10] as developed in the original reports on ESMs [15], [16] to divide the Chinese participants into two groups (ESMs and “Norms”). While consistent with the previous literature, it might be argued that we have dichotomised what is continuous, producing groups where there is in fact a continuum. However, as plotted in Figure 2, the data suggest that the 30% ES criterion does capture something that other parameters (eg median prosaccade latency) do not. We reported previously that the distribution of %ES in Caucasian and Chinese groups is different (Figure 5 in [10]), with no occurrence in the Caucasian data of the bulge that appears to the right of the criterion line in Figure 2B. The data do not appear to be consistent with the hypothesis that the distribution of this parameter is simply shifted to the right in the Chinese group as a whole. There continue to be many Chinese participants who in overlap conditions execute few ES, who are indistinguishable from their Caucasian counterparts. The difference between the two populations is the occurrence of the high number of participants in the Chinese population who execute many ES in prosaccade overlap conditions, defined here as ESMs.

While the 30% criterion is to some extent arbitrary, it is not equivalent to other procedures such as performing a median split on a continuous variable. In fact the median proportion of ES for our dataset of 76 participants is 15%. The higher 30% criterion was originally adopted because it identified a particular type of naïve participant [15]. Our main aim in the current experiment was to investigate how participants meeting this criterion behave in a voluntary saccade task (the antisaccade task).

The difference between the ESMs, defined as discussed above, and other participants was not a simple speeding of saccade reaction times. There was considerable overlap between the groups in terms of their median saccade latencies (Figure 2A). However, average frequency distribution histograms (Figure 1) demonstrated an alteration of the relative proportions of saccades within latency ranges falling close to those suggested previously for express, fast and slow regular saccades [24]. The ESMs exhibited both a selective overproduction of express saccades, and a complimentary underproduction of saccades of longer latency. The distributions also confirmed that a latency range of 80 ms to 130 ms captured the “express peak” previously encountered infrequently in healthy naïve participants in prosaccade overlap tasks [24], [26]. In addition, the non-overlapping 95% confidence intervals suggest that the criterion we have used produces a clear separation of the two groups. The normal group exhibited saccade distributions essentially indistinguishable from those reported in many other studies.

Our second objective was to compare ESM and normal participants, defined on the basis of their performance in the prosaccade task, using the antisaccade task [23], [27]. Antisaccades require the inhibition of responses towards a suddenly appearing target (an error prosaccade), the transformation of the stimulus position into a voluntary motor command, and the execution of a voluntary saccade (the correct antisaccade) to the mirror image position of the stimulus. Antisaccade directional error rate was higher in the ESMs (41±24%) compared to the normal subjects (28±16%). While the distribution of correct antisaccade latency for ESM and normal groups was identical, a large express peak persisted in the ESM error prosaccade latency distribution. We also observed, that in the ESMs there was a slight asymmetry in the directional error rate; the rate tended to be higher when the target appeared to the right of fixation. This may be related to the production of ES, for which there is also a slight asymmetry, with more ES generated with targets on the right.

Directional error rates vary between studies, and are affected by diverse methodological issues such as prior participant experience, task instructions, target eccentricity and number [27], [28]. We used consistent methods and instructions across all participants and counterbalanced the order of overlap and antisaccade runs. We were explicit about instructing participants not simply to look in the opposite direction to the target, but to look to the mirror image position of the target. A synchronous antisaccade task was used (the saccade target appeared when the fixation target was extinguished) as opposed to a gap antisaccade task which encourages both the production of lower latency responses and express saccade errors [29]. An error rate of 23±17% for a synchronous task (albeit with targets appearing at a range of eccentricities from 2° to 10°) for a large sample of over 2000 young men aged between 18 y and 24 y has been reported previously [30]. This compares reasonably well with the error rate in our normal participants (28±16%). Given the variability in directional error rate in this and in many other studies, how should we interpret the difference we observed? A complementary approach is to calculate the difference as an “effect size”. Often this is the effect size of an intervention (eg the comparison of some measurement in a treated group versus the same measurement in an untreated group). Here we compared the directional error rate in the ESM and normal groups using Cohen’s d [31], [32]. The computed “effect size” (ie the standardised magnitude of the difference in error rate) was 0.69; this is considered to be a medium to large effect size [31], [32].

Evdokimidis et al [30] plotted pooled latency distributions for both correct antisaccades and error prosaccades for their large sample. For correct antisaccades they showed a broad unimodal distribution with a mean (±SD) of 270±39 ms, similar to what we observed in both ESM (290±37 ms ) and normal groups (290±61 ms; Figure 5A,C). Their distribution of error prosaccades was also unimodal (mean 208±38 ms) with no responses at a latency of less than 120 ms. This is broadly similar to what we observed in normal participants (with regard to both the mean and variability; Figure 5B), but very different to the ESMs, where we observed both a large peak in the express range and a reduction in the proportion of saccades with longer latencies (Figure 5D). The second peak in the ESM distribution is at 160 ms, precisely where the main peak is in the distribution for the normal participants. While in the normal participant distribution there were some errors in the express range (11±11%), the proportion was much smaller than in the ESMs (36±26%). It is the generation of this population of express saccades that produces the overall statistically significant reduction in error prosaccade latency in the ESMs, not a generalised shift of a unimodal distribution to the left. Thus, in circumstances where participants would be expected to inhibit reflexive responses, and in which they are aided by the use of a synchronous task rather than a gap task, ESMs continue to exhibit high proportions of express saccades.

There were a number of relationships between parameters in the prosaccade and antisaccade tasks. Participants with lower median prosaccade saccade latency tended to be those with higher antisaccade directional error rates (Figure 6A) and lower antisaccade error prosaccade latency (Figure 6C). However, as discussed above, the ESMs exhibit a selective overproduction of saccades within a particular latency range, not a general reduction in saccade latency. Therefore median latency in the prosaccade task poorly captures the difference between groups, with considerable overlap between normal and ESM participants. Not surprisingly when the data are plotted using the percentage of express saccades in prosaccade tasks, a clearer pattern emerges (Figure 6B,D). In common with other studies in which these various relationships have been examined, the correlations, while statistically significant, were relatively weak [33], [34]. The highest correlation coefficient was for the relationship between the percentage of express saccades in prosaccade tasks and pro-saccade errors in the antisaccade task. This is consistent with the hypothesis that pro-saccade errors in the antisaccade task are uninhibited reflexive responses to the target. ESMs were relatively unsuccessful at inhibiting reflexive responses (hence the higher directional error rate), but as will be discussed below this could be a consequence of the low latency nature of their reflexive responses.

A number of models have been developed which seek to explain patterns of saccade latency. Accumulator models describe saccade triggering in terms of a decision signal rising from a baseline to a threshold; when the threshold is crossed, a saccade is initiated [35]. Saccade latency modulations are related to changes in the baseline level of activity, the rate of rise of the decision signal or the level of the threshold [36]. For antisaccades, competitive race accumulator models [37] assume that a prosaccade decision signal (initiated by the appearance of the target, and therefore exogenously triggered) and an antisaccade decision signal (generated internally and therefore usually considered to be endogenous) “race” each other towards threshold. If the prosaccade signal “wins”, a prosaccade error is the result; if the antisaccade signal “wins”, a correct antisaccade is the result. On this account it is the inability of ESMs to successfully inhibit low latency prosaccades (many in the express latency range) that explains their performance in antisaccade tasks (higher error rates, lower prosaccade error latencies), the close similarity between the average distributions for the prosaccade task (eg Figure 1D) and the antisaccade prosacccade error distributions (Figure 5D), and the correlation between the percentage of express saccades in prosaccade and antisaccade tasks. The dissimilarity in the ESM distributions (higher express peak, and lower subsequent peaks in prosaccade compared to prosaccade error distributions) could be explained by our using a synchronous rather than overlap antisaccade task.

One appealing aspect of accumulator models in general, and race models in particular, is that various model elements appear to map to specific saccade-related neurophysiological structures and processes in a relatively straightforward manner [23], [38]. Indeed the underlying neurophysiology, particularly with respect to the superior colliculus is a key component of some models [39], [40]. The rate of rise of activity in saccade related neurons in both frontal eye fields (FEF) [41] and the superior colliculus (SC) [13], [42] is related to saccade latency. There is some evidence that the trigger threshold is relatively fixed [41], [43], while pretarget activity levels vary systematically in both FEF [44]and SC [13]. Compared to pretarget activity levels in both the FEF and SC when a normal latency saccade is executed, pretarget activity is increased prior to an express saccade, leading to the visual response in the SC being sufficient to trigger a saccade [12], [13]. Pretarget activity levels in both the SC and FEF are also relatively higher in prosaccade errors relative to correct antisaccades [44], [45]. In both cases, the behavioural result (an express saccade or prosaccade error) is explained in terms of increased pretarget activity in the SC; the decision signal rises from a higher baseline, and assuming a constant rate of rise and threshold, crosses threshold triggering a saccade earlier than would otherwise be the case. However, note that in the antisaccade task, a general increase in pretarget activity in the SC in ESMs, would imply higher antisaccade directional error rates and decreased prosaccade error latencies (which we observed) *and* decreased antisaccade latencies (which we did not observe). A change in trigger threshold at the level of the SC would also imply linked alteration in both error prosaccades and correct antisaccades. Rather, our results suggest that in ESMs there is a specific alteration in the timing and variability of reflexive prosaccades, perhaps related to descending inhibitory control, which compromises their performance in the antisaccade task.

Descending inhibitory control in the oculomotor system involves a number of cortical areas (FEF; dlPFC; supplementary eye fields; parietal eye fields). Frontal lesions in humans have been shown to increase antisaccade directional error rates [46], with most recent attention directed at dlPFC [47], [48]. However, effects on saccade, prossacade error and antisaccade latency were either not reported in these studies, or the results were unclear. Increased directional error rates have been produced in healthy humans using TMS of the dlPFC [49], and deactivation of dlPFC in monkeys using cooling [50]. However, microstimulation of dlPFC has also been shown to increase error rates, and increase saccade latency [51]. Given this background, it would be unwise to claim that a specific alteration in dlPFC can explain the pattern of results observed in the ESMs [52]. However, a specific and circumscribed alteration of frontal function in the ESMs, affecting inhibition of reflexive responses, leading to poorer performance in the antisaccade task, might explain our observations.

Given that in ESMs a high proportion of express saccades persists even in a testing paradigm which requires high levels of inhibition, it seems likely that they may also persist in other task contexts [53]. Thus in comparing Chinese and other participant groups (in which ESMs remain rare) using a range of oculomotor based measures, a low level effect (high numbers of express saccades in a large proportion of Chinese participants) not related to task context might bias dependant measures and be mistakenly assumed to be driven by cultural differences between groups. For example, Chinese groups have been observed to make more fixations and have shorter fixation times in scene processing tasks, compared to non-Chinese participants [19], [20]. But suppose that a large proportion of the Chinese participants executed the equivalent of express saccades while scanning scenes (presumably sequences of saccades with short fixation durations), this might increase the average number of saccades and reduce average fixation duration compared to a group that did not contain a high proportion of ESMs.

The cultural neuroscience literature assumes that environment (broadly constructed to include everything from the physical environment to the general cultural milieu) acts on a basically similar neurophysiological substrate to produce observed differences between population groups (ie distinct cultural groups). Our results demonstrate a difference in reflexive oculomotor control within a particular cultural group. While that there were undoubtedly many ways in which the habits and environments of our individual participants differed, it is difficult to see how these might produce the specific alteration in saccade function we have observed. Our results could imply a difference in neurophysiological substrate, at least as far as eye movement control is concerned, not primarily related to culture.

It has recently been demonstrated that for certain relevant functions such as attentional processing [8] and eye movement strategies during face processing [9] ethnicity (or at least continent of origin) rather than culture is a key determinant of performance. For the difference between Chinese and non-Chinese groups we have observed, it remains to be established whether the high proportion of ESMs persist in Chinese groups whose main cultural exposure is non-Chinese.

## Supporting Information

Figure S1 **Individual frequency distribution histograms of latency for the 16 ESMs.** In each plot the median prosaccade latency, and the percentage of express saccades is shown. Plots are ordered by %ES, from highest (top left) to lowest (bottom right).
(TIF)

Figure S2 **Individual frequency distribution histograms for 60 normal participants.** Conventions as for Figure S1.
(TIF)
