# Correction: Performance Deficits in a Voluntary Saccade Task in Chinese “Express Saccade Makers”

**DOI:** 10.1371/annotation/98b3e349-5e61-47a0-a8e5-ebcd6940da21

**Published:** 2012-11-07

**Authors:** Paul C. Knox, Nabin Amatya, Xiaoyu Jiang, Qyong Gong

The fourth author's name was spelled incorrectly. The correct name is: Qiyong Gong.

