# Supplementary figures and images for: Performance Deficits in a Voluntary Saccade Task in Chinese “Express Saccade Makers”
Source: PLoS One. 2012 Oct 16;7(10):e47688. doi: 10.1371/journal.pone.0047688 (PMC3472994; doi:10.1371/journal.pone.0047688)

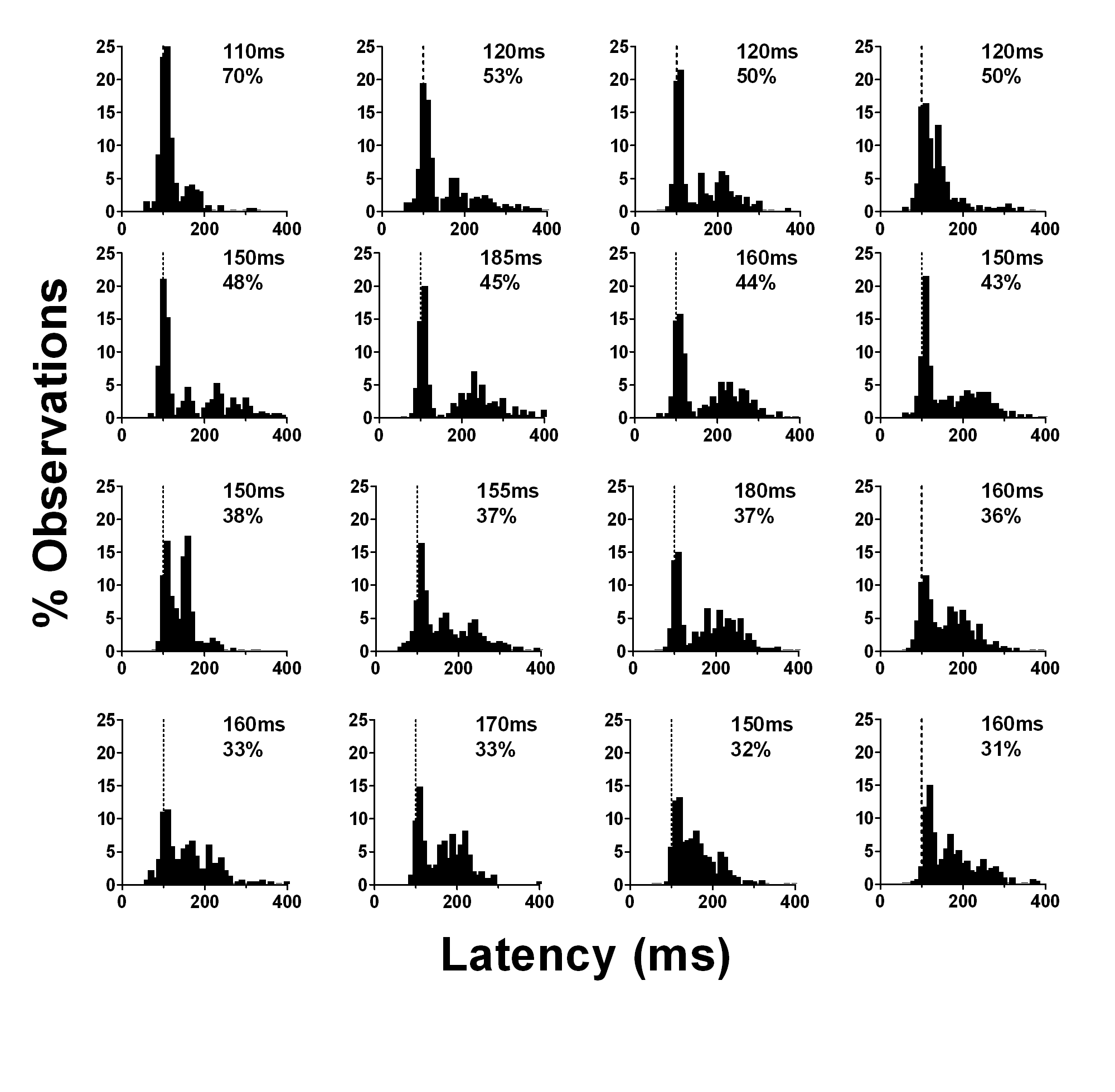

Supplement: Figure S1 — Individual frequency distribution histograms of latency for the 16 ESMs. In each plot the median prosaccade latency, and the percentage of express saccades is shown. Plots are ordered by %ES, from highest (top left) to lowest (bottom right). (TIF) [file pone.0047688.s001.tif]

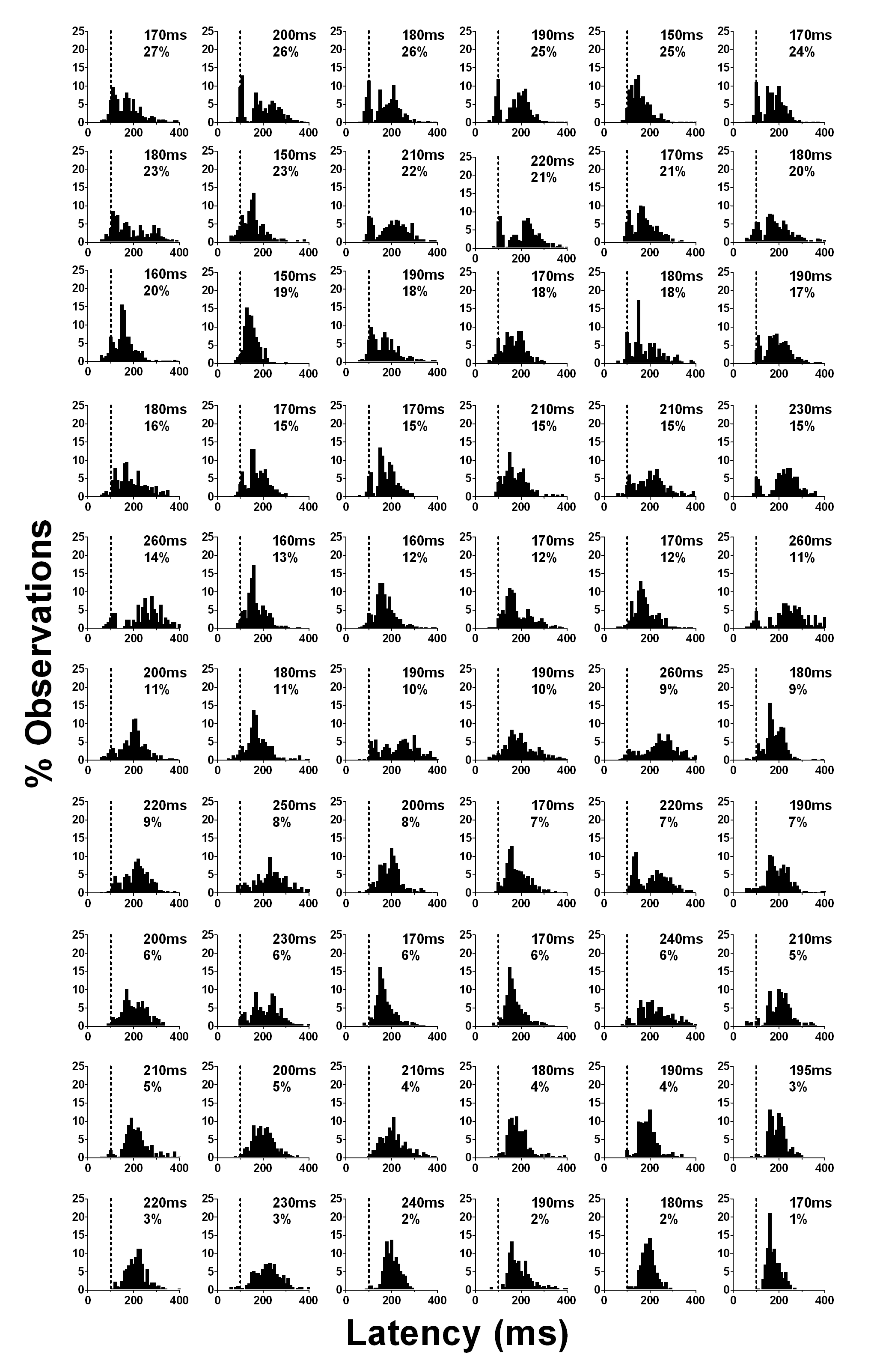

Supplement: Figure S2 — Individual frequency distribution histograms for 60 normal participants. Conventions as for Figure S1. (TIF) [file pone.0047688.s002.tif]
